# Supplementary material for: Comprehensive evaluation of coding region point mutations in microsatellite‐unstable colorectal cancer
Source: EMBO Mol Med. 2018 Aug 14;10(9):e8552. doi: 10.15252/emmm.201708552 (PMC6402450; doi:10.15252/emmm.201708552)
Supplement: Supplementary file 1 — Appendix [file EMMM-10-e8552-s001.pdf]

COMPREHENSIVE EVALUATION OF CODING REGION POINT MUTATIONS IN MICROSATELLITE UNSTABLE COLORECTAL CANCER

Appendix material

Johanna Kondelin[1,2], Kari Salokas[3,4], Lilli Saarinen[2], Kristian Ovaska[2], Heli Rauanheimo[1,2], Roosa-Maria Plaketti[1,2], Jiri Hamberg[1,2], Xiaonan Liu[3,4], Leena Yadav[3,4], Alexandra E. Gylfe[1,2], Tatiana Cajuso[1,2], Ulrika A. Hänninen[1,2], Kimmo Palin[1,2], Heikki Ristolainen[1,2], Riku Katainen[1,2], Eevi Kaasinen[1,2], Tomas Tanskanen[1,2], Mervi Aavikko[1,2], Minna Taipale[5], Jussi Taipale[1,2,6,7], Laura Renkonen-Sinisalo[8], Anna Lepistö[8], Selja Koskensalo[9], Jan Böhm[10], Jukka-Pekka Mecklin[11,12], Halit Ongen[13,14,15], Emmanouil T. Dermitzakis[13,14,15] Outi Kilpivaara[1,2], Pia Vahteristo[1,2], Mikko Turunen[2], Sampsa Hautaniemi[2], Sari Tuupanen[1,2], Auli Karhu[1,2], Niko Välimäki[1,2], Markku Varjosalo[3,4], Esa Pitkänen[1,2], Lauri A. Aaltonen[1,2]

1 Medicum/Department of Medical and Clinical Genetics, University of Helsinki, 00290 Helsinki, Finland
2 Genome-Scale Biology Research Program, Research Programs Unit, University of Helsinki, 00290 Helsinki, Finland
3 Institute of Biotechnology, University of Helsinki, 00014 Helsinki, Finland
4 Helsinki Institute of Life Science, University of Helsinki, 00014 Helsinki, Finland
5 Department of Medical Biochemistry and Biophysics (MBB), Division of Functional Genomics, Karolinska Institutet, 17177 Stockholm, Sweden
6 Department of Biosciences and Nutrition, Karolinska Institutet, 141 57 Huddinge, Sweden
7 Science for Life Center, 17121 Huddinge, Sweden
8 Department of Surgery, Helsinki University Central Hospital, Hospital District of Helsinki and Uusimaa, 00290 Helsinki, Finland
9 The HUCH Gastrointestinal Clinic, Helsinki University Central Hospital, 00290, Helsinki, Finland
10 Department of Pathology, Jyväskylä Central Hospital, 40620 Jyväskylä, Finland
11 Department of Surgery, Jyväskylä Central Hospital, University of Eastern Finland, 40620 Jyväskylä, Finland
12 Department Sport and Health Sciences, University of Jyväskylä, 40620 Jyväskylä, Finland
13 Department of Genetic Medicine and Development, University of Geneva Medical School, 1205 Geneva, Switzerland
14 Institute for Genetics and Genomics in Geneva (iGE3), University of Geneva, 1211 Geneva, Switzerland
15 Swiss Institute of Bioinformatics, 1211 Geneva, Switzerland

APPENDIX FIGURES

Appendix Figure S1
Appendix Figure S2
Appendix Figure S3
Appendix Figure S4
Appendix Figure S5

APPENDIX TABLES

Appendix Table S1
Appendix Table S2

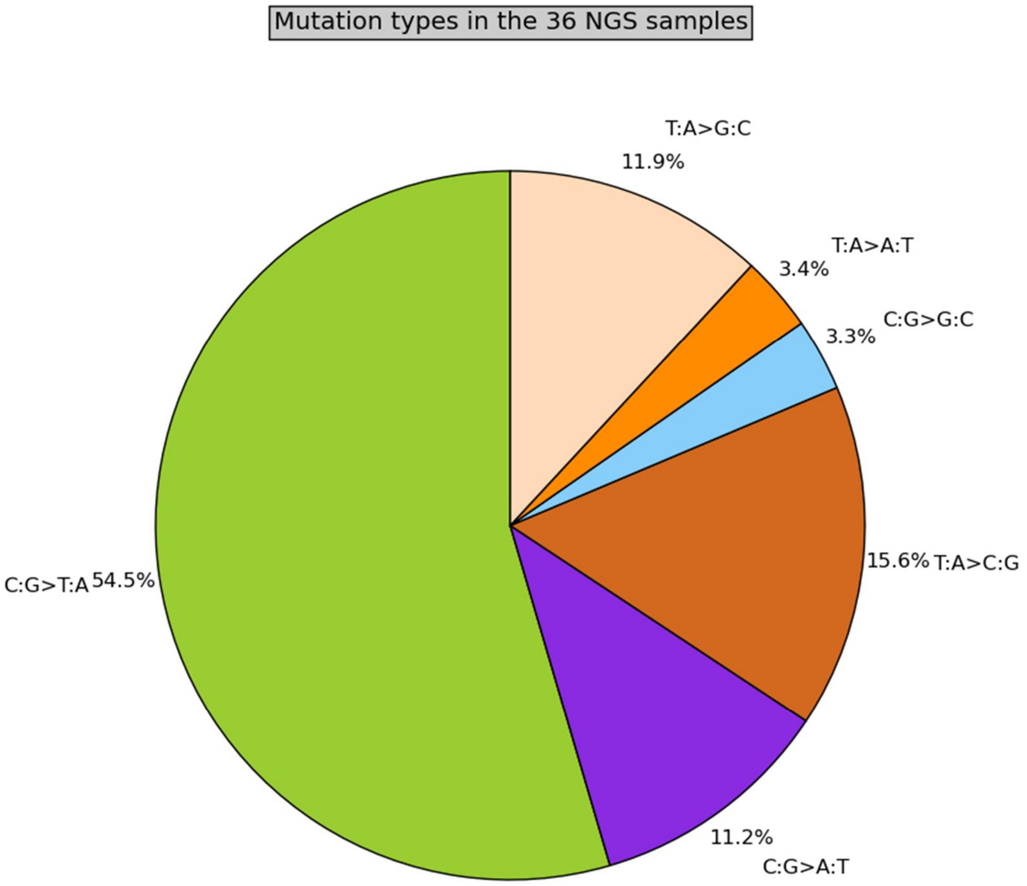

Appendix Figure S1. Mutation types NGS. The fractions of different mutation types in the 36 NGS samples.

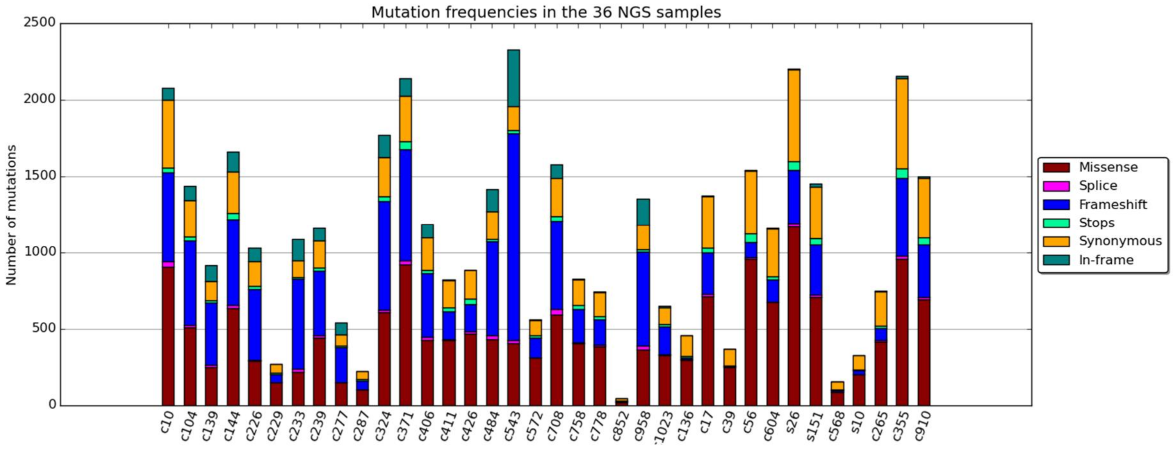

Appendix Figure S2. **Mutation frequencies in the 36 NGS samples.** ‘Stops’ includes both stop-gain and stop-loss mutations.

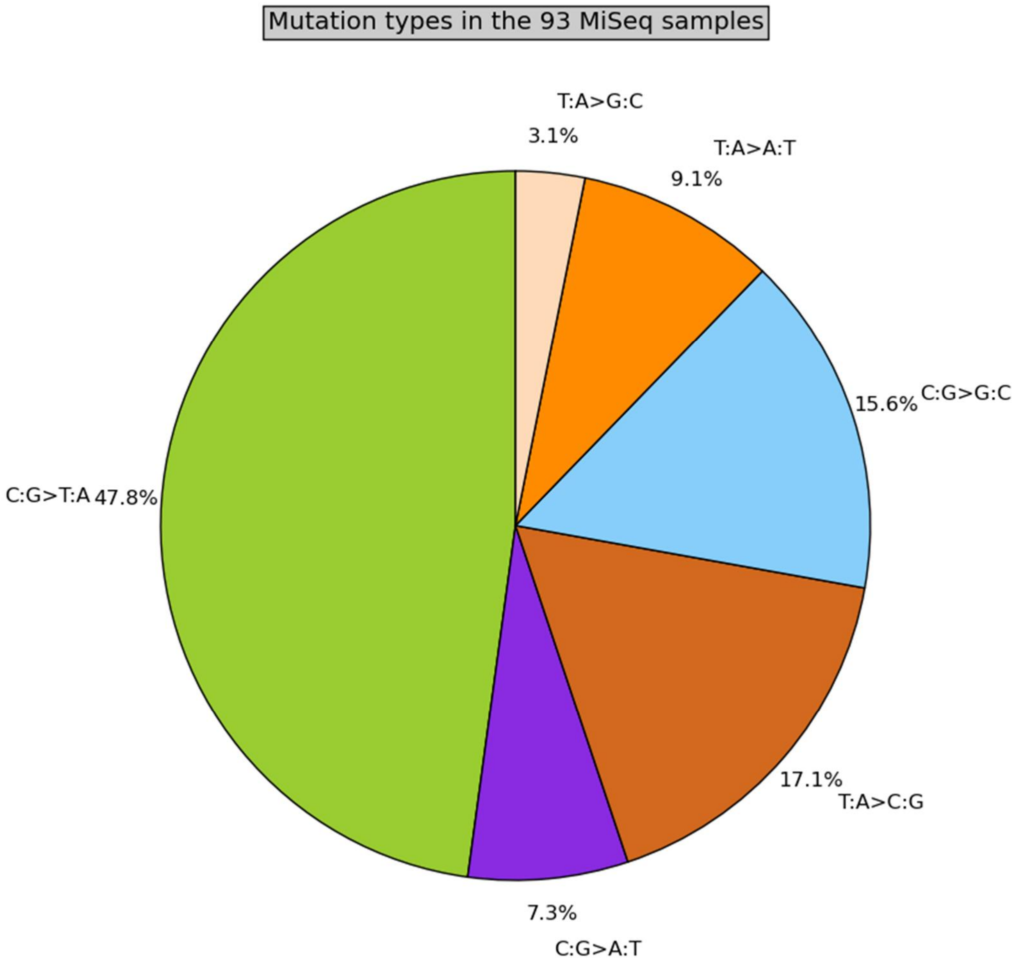

Appendix Figure S3. The fractions of different mutation types in the 93 MiSeq samples.

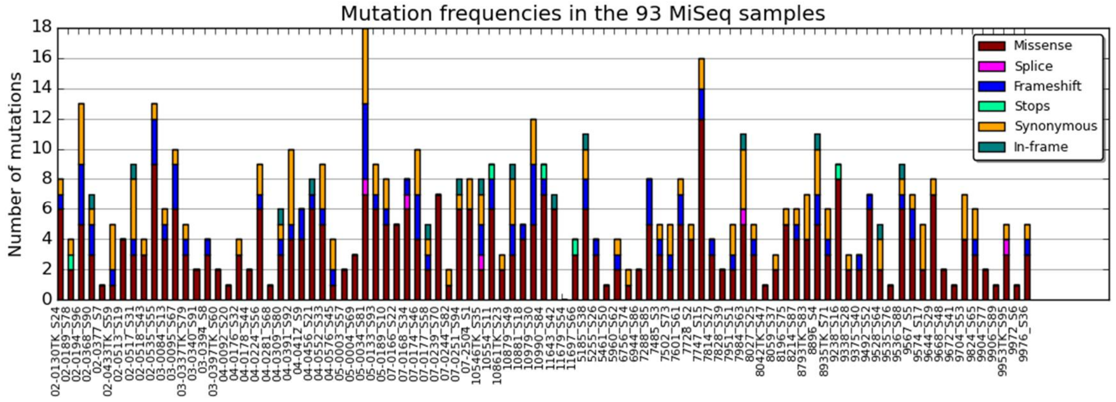

Appendix Figure S4. Mutation frequencies MiSeq. Mutation frequencies in the 93 MiSeq samples. ‘Stops’ includes both stop-gain and stop-loss mutations.

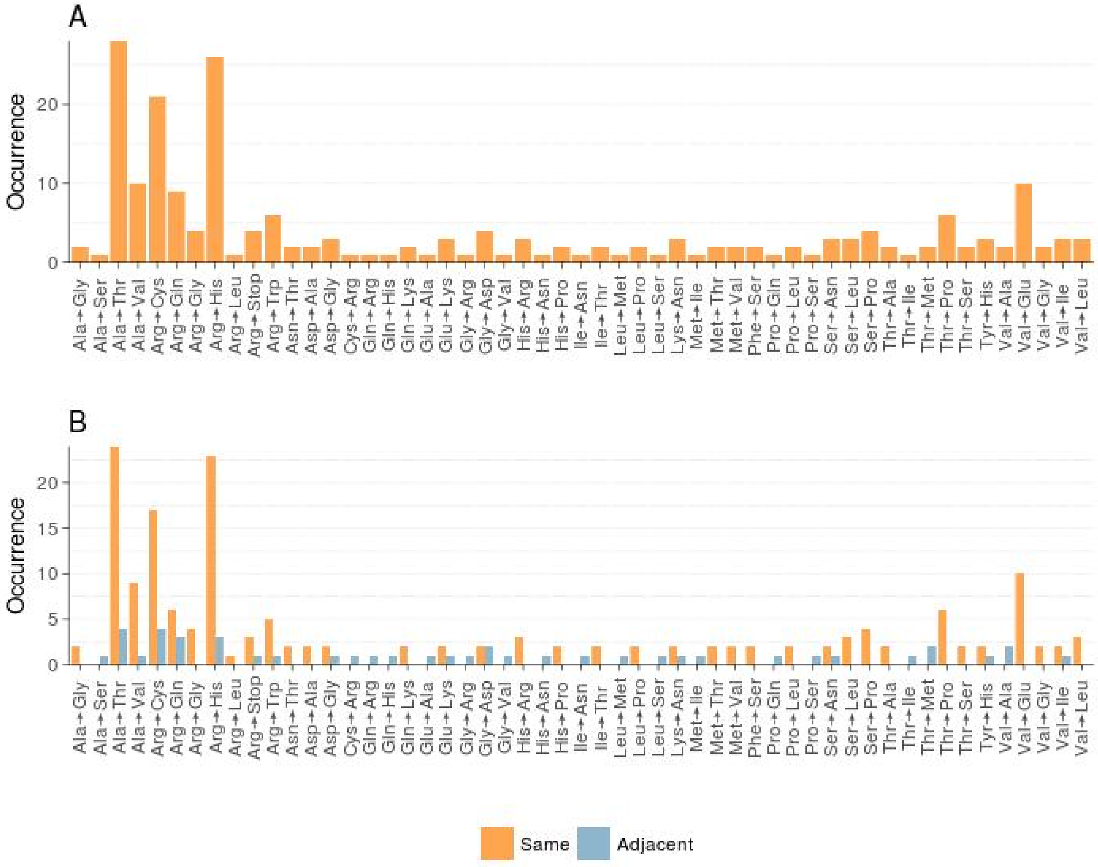

Appendix Figure S5. Amino acids targeted. A) The number of all the amino acid substitutions in the 97 hot spots consisting of non-synonymous mutations targeting either the same codon or adjacent codons in at least two samples. B) The number of all the amino acid substitutions in the 75 mutation hot spots consisting of non-synonymous mutations targeting the same codon (yellow), and in the 22 mutation hot spots consisting of non-synonymous mutations targeting adjacent codons (blue).

Appendix Table S1. Clinical information of the 22 RNA sequenced normal tissues of CRC patients

Sample series	Sample number	Source of normal tissue	Sex
c	100	intestine	M
c	135	intestine	M
c	206	intestine	F
c	263	intestine	F
c	279	intestine	F
c	281	intestine	M
c	352	intestine	M
c	402	intestine	M
c	439	intestine	F
c	479	intestine	M
c	497	intestine	F
c	540	intestine	M
c	571	intestine	M
c	581	intestine	F
c	619	intestine	M
c	655	intestine	M
c	775	intestine	M
c	897	intestine	M
c	1019	intestine	F
s	56	intestine	F
s	146	intestine	F
s	204	intestine	M

Appendix Table S2. Mutation effects defined for MutSigCV

Description	Effect
Nonsynonymous substitution	nonsilent
Splice site 1 mutation	nonsilent
Splice site 2 mutation	nonsilent
Other splice site mutation	noncoding
Stop loss mutation	nonsilent
Stop gain mutation	nonsilent
Synonymous mutation	silent
Transcription start site mutation	noncoding
