# Supplementary material for: Comprehensive evaluation of coding region point mutations in microsatellite‐unstable colorectal cancer
Source: EMBO Mol Med. 2018 Aug 14;10(9):e8552. doi: 10.15252/emmm.201708552 (PMC6402450; doi:10.15252/emmm.201708552)
Supplement: Supplementary file 2 — Expanded View Figures PDF [file EMMM-10-e8552-s002.pdf]

Expanded View Figures

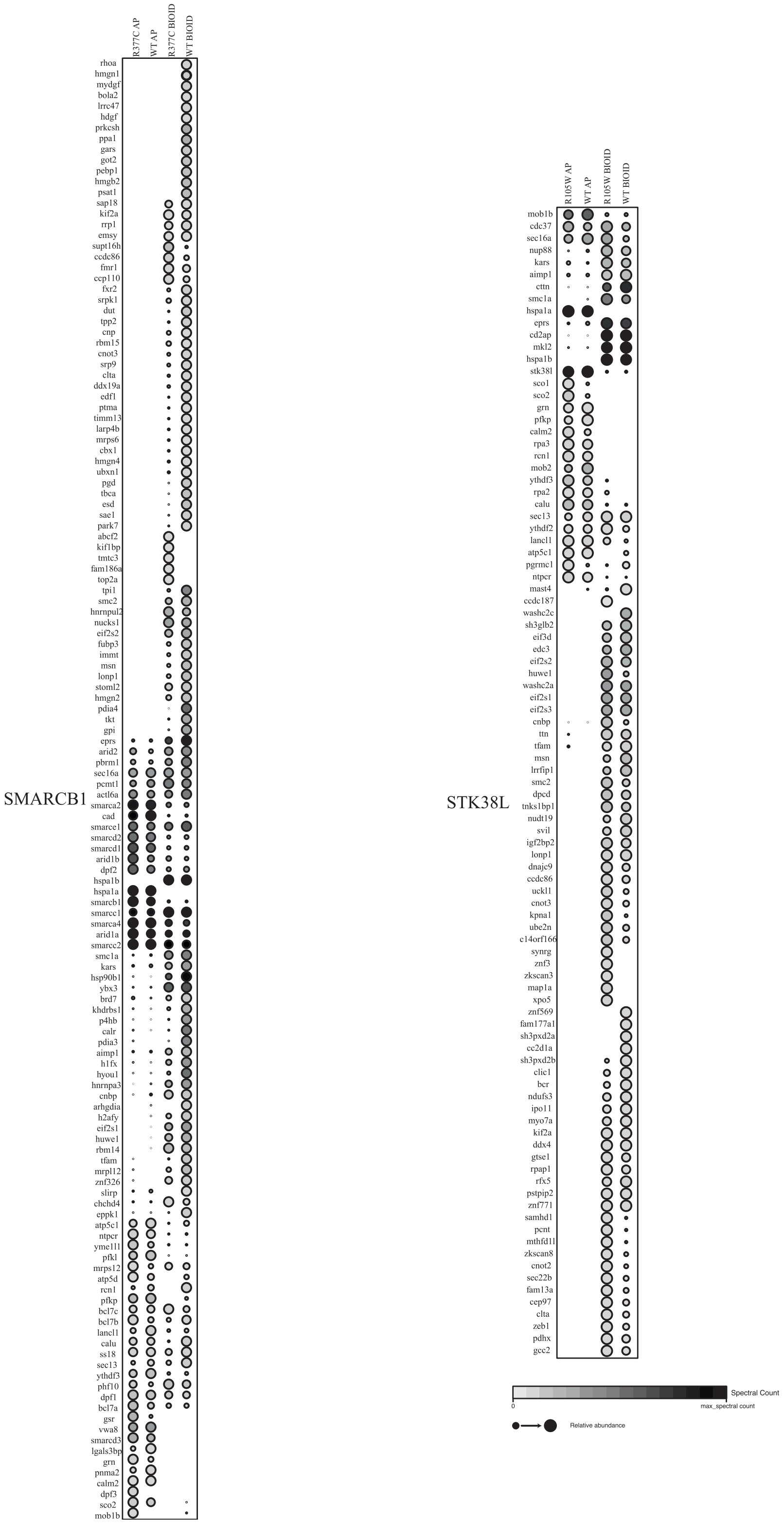

Figure EV1. Dot blot visualization of the STK38L and SMARCB1 high-confidence interactions.

The AP-MS and BioID results of the SMARCB1 wild type and the R377C mutant, and the STK38L wild type and the R105W mutant after filtering, yielding only the high-confidence interactors.

© 2018 The Authors

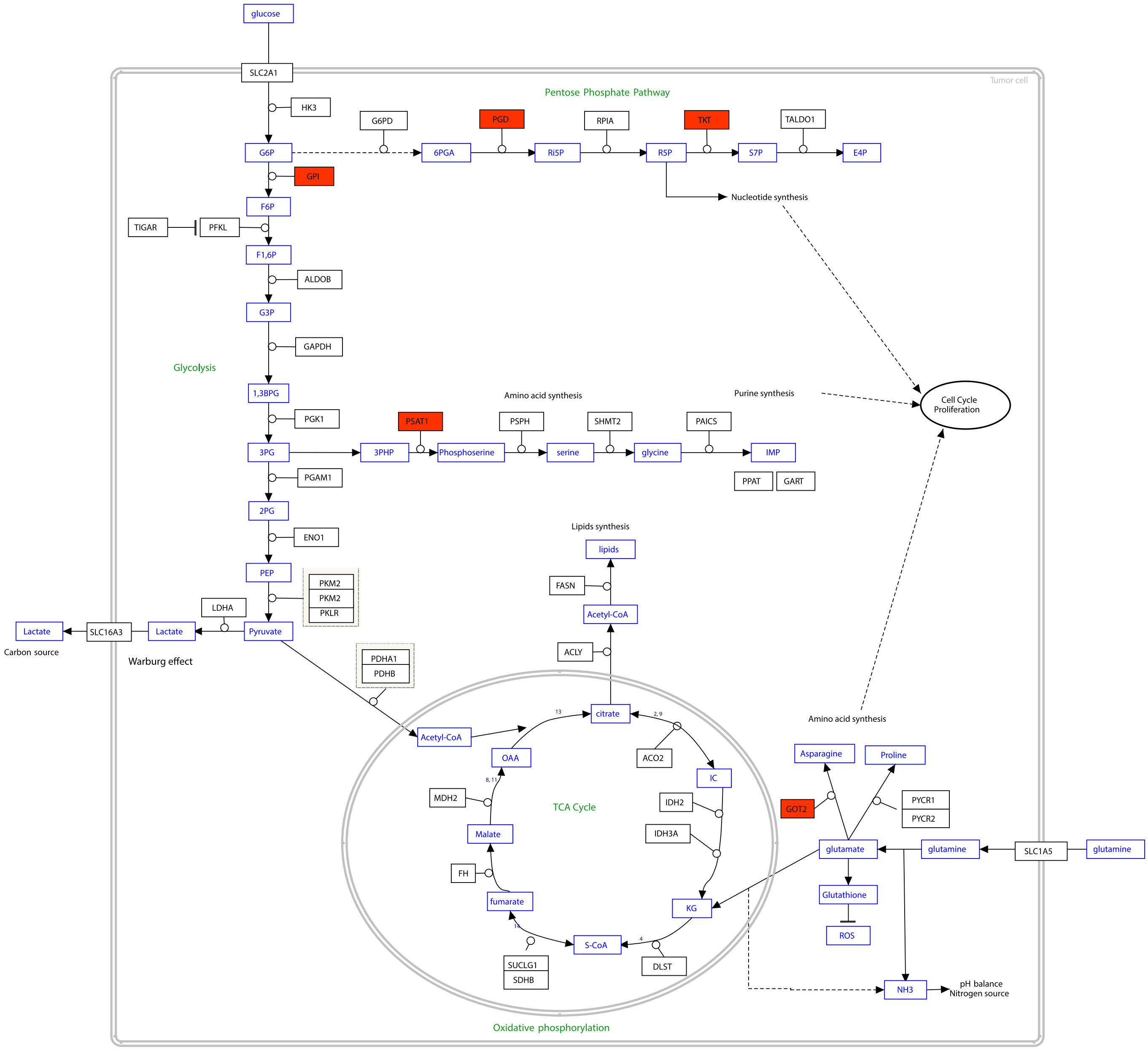

Figure EV2. SMARCB1 interacts with several proteins of the "Metabolic Reprogramming in Colon Cancer" pathway.

The analysis of the SMARCB1 wild type and the R377C mutant led to the identification of 52 altered HCIPs. Of these, five proteins (shown in red fill) associated with the "Metabolic Reprogramming in Colon Cancer" pathway.

 © 2018 The Authors

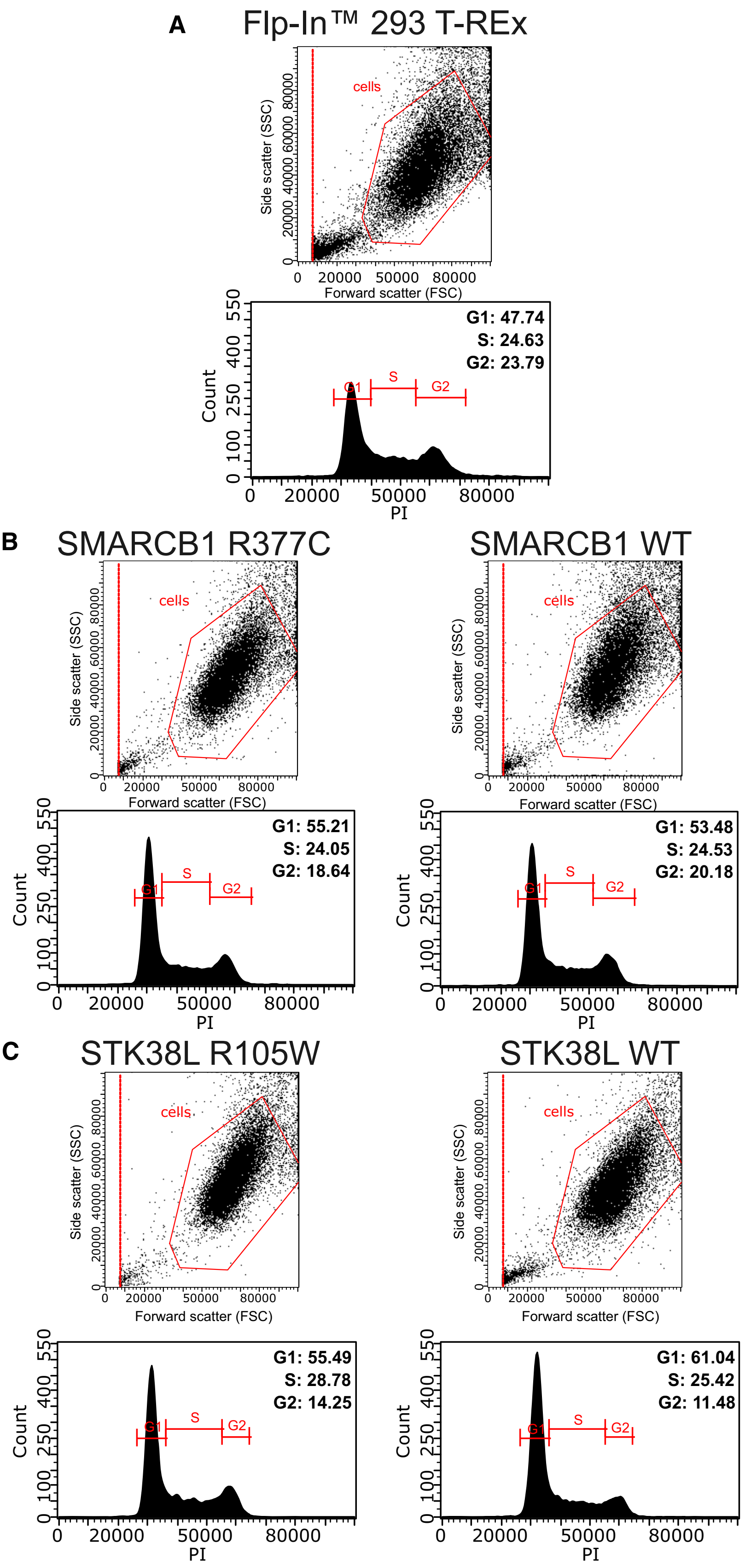

Figure EV3. Cell cycle analysis using FACS.

A–C The corresponding transgene expressing Flp-In T-REx 293 cells was fixed, processed, and analyzed according to the analysis protocol. The gated cells in the scatter plots (SSC versus FCS) were selected, and the corresponding cell cycle profiles are shown for each of the cell lines (A–C). The percentage of cells in each cell cycle phase (G1, S, and G2) are calculated from the PI-area.

© 2018 The Authors
